# Mineral substrates as evolutionary drivers of soil microbial diversity through the rare biosphere

**DOI:** 10.1128/aem.02011-25

**Published:** 2025-12-09

**Authors:** Beibei Wang, Jianchao Zhang, Xiangyu Zhu, Yuebo Wang, H. Henry Teng

**Affiliations:** 1 School of Earth System Science, Institute of Surface-Earth System Science, Tianjin University, Tianjin, China; Colorado School of Mines, Golden, Colorado, USA

**Keywords:** mineral-microbe interaction, community assembly, community evolution, rare taxa, mineralosphere

## Abstract

Minerals are fundamental yet underappreciated drivers of microbial ecology. Traditionally viewed as passive nutrient sources or inert scaffolds, their broader ecological roles remain poorly defined. This study investigates the evolutionary influence of substrates (minerals and rocks) on soil bacterial communities through serial passage evolution experiments. Soil-derived microbial consortia from three distinct locations were exposed to nutritive (olivine, granite, diorite) and non-nutritive (quartz, kaolinite, montmorillonite) substrates under nutrient-rich conditions to isolate substrate-specific effects. Results revealed systemic variations of community structure across all treatments, characterized by elevated *Firmicutes*/*Bacteroidetes* ratio and taxonomic changes predominantly driven by rare taxa. These discoveries indicate that, under the influence of substrates, the communities shifted toward ones that preferentially utilize more labile carbon. Crucially, the acute responsiveness of rare taxa to mineral-induced environmental selection suggests that, although abundant taxa appeared to maintain core community functions, the rare biosphere facilitated niche specialization and functional diversification. These findings position minerals as dynamic drivers of microbial ecology and evolution, highlighting the mineralosphere as a critical microhabitat where abiotic properties govern biodiversity, functional redundancy, and evolutionary innovation in soil ecosystems.

**IMPORTANCE:**

Even under nutrient-rich conditions, non-nutritive and chemically inert minerals, exemplified by quartz, actively reshape microbial community assembly. Through controlled serial-passage experiments, we show that distinct substrates selectively enrich rare biosphere members that expand functional potential and seed adaptation, while dominant taxa sustain core processes. These results reveal that mineral surface properties and physical interfaces, rather than nutrient supply, govern microbial diversification and evolutionary trajectories. Accordingly, the mineralosphere emerges as a dynamic microhabitat where abiotic complexity regulates biodiversity, metabolism, and long-term community succession. This reframes minerals and rocks as active ecological and evolutionary agents, bridging geomicrobiology and evolutionary ecology, with implications for soil health, biogeochemical cycling, and the origin and maintenance of microbial diversity.

## INTRODUCTION

Soil microbial communities govern ecosystem dynamics by driving processes such as nutrient cycling, organic matter decomposition, and carbon sequestration. These communities, comprising bacteria, fungi, archaea, and viruses, are shaped by a complex interplay of environmental variables across varying spatial and temporal scales (1–3). Among these variables, soil pH, nutrient availability, and climate are established primary drivers regulating community composition (3–5), metabolic and functional diversity (2, 5, 6), and assembly richness and biogeographic distribution (2, 3, 5).

A ubiquitous yet often overlooked environmental factor is that of rocks and minerals (collectively termed substrates). These geomaterials are integral components of soils, but their influences on microorganisms are frequently eclipsed by fast-acting variables such as water availability. Traditional view holds that minerals regulate microbial growth over time by providing nutrient and/or energy elements (7–11). Non-nutritive minerals, albeit capable of providing physical scaffolds for colonization (12), are conventionally considered non-critical for microbial development. However, evidence abounds suggesting the oversimplification of this approach. Early studies noted that non-nutrient materials like chalk and kaolin promote bacterial growth (13), presumably due to the presence of sorbed nutrients at the grain surfaces. However, recent data revealed that certain microbial taxa show preferences for nutrient-poor minerals like quartz (14, 15) but are suppressed by Fe-bearing solids of low crystallinity (16). Studies also documented complex relationships between minerals and microorganisms. For instance, while clays are known to protect bacteria from predation (17–19), they can limit cell mobility (20) to restrain microbial access to organic matter (21). These advances ultimately led to the conceptualization of “mineralosphere”—microenvironments around mineral particles where mineralogical properties play determinant roles in modulating microbial community dynamics, nutrient cycling, and evolutionary processes (22, 23). Nevertheless, regardless of specific mechanisms of cell-mineral interactions and the nutrient/energy values of substrates, the effect of geological substances on microbial growth is expected to be minimal if the system is not subject to nutrient and/or energy stress (24).

This study attempts to examine the evolutionary impact of substrates on soil bacterial communities using a serial passage evolution experiment designed to simulate natural selection processes that drive microbial adaptive evolution over multiple generations. The experiment exposes soil-derived microbial consortia from three distinct locations in northeastern China to nutritive (olivine, granite, diorite) and non-nutritive (quartz, kaolinite, montmorillonite) substrates under nutrient-rich culture conditions. This approach isolates the effects of substrate composition and structure on microbial growth while minimizing confounding environmental stresses. The specific research goal is to determine how substrate-mediated selection drives microbial adaptation and evolution with emphasis on community composition, structure, and functions. We hypothesized that (i) each substrate would selectively enrich a distinct microbial assemblage and (ii) sustained exposure to substrates would drive community compositional shifts and possibly evolutionary changes. We will pay special attention to rare microbial taxa (low abundance species) as these organisms are potentially more sensitive to environmental shifts due to their niche specificity (25–28). Through testing these hypotheses, we intend to establish a framework to delineate how minerals and rocks influence microbial development and ecology via their physicochemical and surface properties.

## RESULTS

### Treatment-induced taxonomic structure change

**Fig 1:**
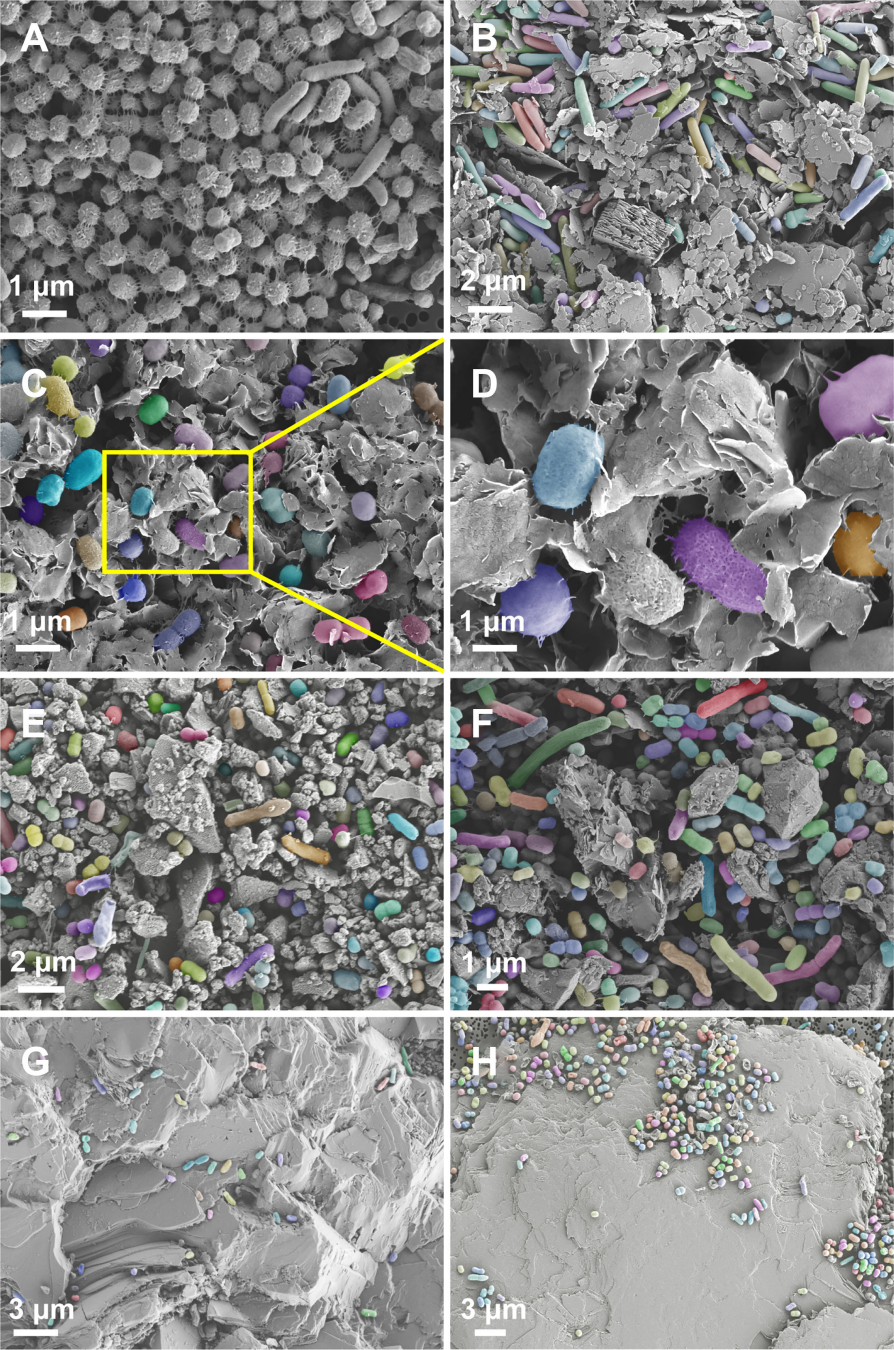
After 50 serial passages in the evolution experiment, the morphology of microbial cells and the modes of microbial–mineral interactions were examined by scanning electron microscopy (SEM). Samples from S2 were selected as a representative for detailed observation. (**A**) CK, no mineral presence; (**B**) kaolinite treatment; (**C** and** D**) montmorillonite treatment; (**E**) olivine treatment; (**F**) diorite treatment; (**G**) granite treatment; (**H**) quartz treatment. Cells in mineral treatments were color-labeled to facilitate visual identification.

Morphological analysis via scanning electron microscopy (SEM) (Fig. 1) showed diverse-shaped cells tightly clustered into aggregates in the CK group (Fig. 1A). In contrast, substrate-treated cells retained intact morphology but exhibited distinct distribution patterns. When incubated with finer mineral/rock particles, cells tended to disperse among grains, forming integrated mineral-cell aggregates (Fig. 1B through F). With larger substrate fragments, cells adhered primarily to particle surface (Fig. 1G and H).

**Fig 2:**
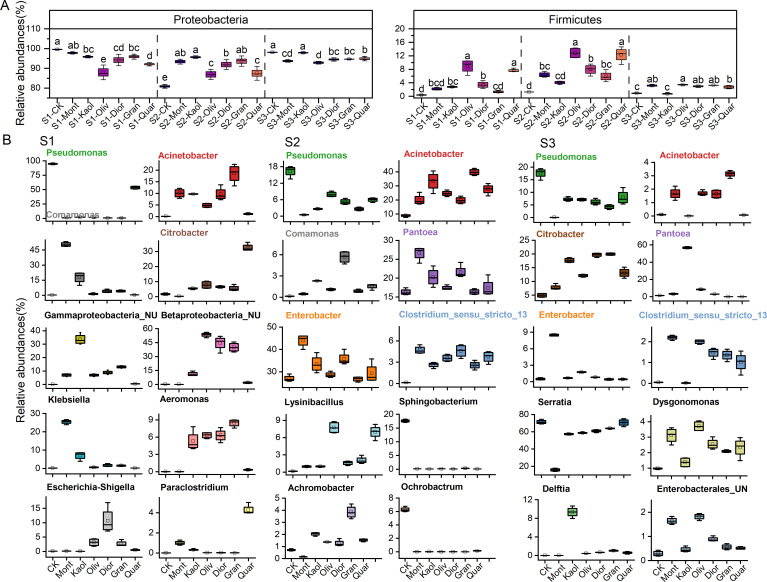
Effects of mineral treatments on the relative abundance of dominant phyla Proteobacteria and Firmicutes (**A**) and top 10 genera (**B**) at each site. Letters in A indicate significant differences within each site (*P* < 0.05. Prior to ANOVA, data normality and homogeneity of variances were tested using the Shapiro–Wilk and Levene’s tests, respectively. When assumptions were not met, non-parametric Kruskal–Wallis tests were used as alternatives. Genera are consistently color-coded, whereas non-overlapping taxa are depicted in black. Columns 1–2 correspond to S1 (Site 1), columns 3–4 to S2 (Site 2), and columns 5–6 to S3 (Site 3). Data are shown as mean  ±  standard deviation of four independent biological replicates (*n*  =  4).

Across all samples (treated or untreated), bacterial communities were dominated by *Proteobacteria* (80%–99%) at the phylum level, followed by *Firmicutes* (0.4%–13%) and *Bacteroidota* (0.01%–17%) (Fig. 2A; Fig. S3). Compared to CK, treatment groups consistently elevated the abundance of *Firmicutes*, with the highest levels observed in Oliv and Quar across all sites. In contrast, substrate effect on *Proteobacteria* varied between sites, increased at S2 but declined at S1 (excluding Mont) and S3 (excluding Kaol) (Fig. 2A; Fig. S3). The site-specific behavior continued at the genus level (Fig. 2B; Fig. S4), with treatments reducing *Pseudomonas* while enriching other genera at S1, limiting *Pseudomonas*, *Sphingobacterium*, and *Ochrobactrum* but promoting *Enterobacter*, *Acinetobacter*, *Pantoea*, and *Clostridium sensu stricto* 13 at S2, and increasing *Clostridium sensu stricto* 13 (except Kaol) at S3.

Besides site-dependent changes in the communities, there also exhibited treatment-specific effect (Fig. 2B; Fig. S4). For instance, Montmorillonite markedly promoted *Comamonas*, *Klebsiella*, and *Paraclostridium* at S1, but selectively favored *Enterobacter* and *Pantoea* while preferentially suppressed *Pseudomonas* and *Achromobacter* at S2. The opposite effects of Mont on *Enterobacter* and *Pseudomonas* also extended to S3, plus an additional highly noticeable retardation to *Serratia*. Kaolinite, another clay substrate, exerted different effect, significantly enriching *Gammaproteobacteria*_unclassified at S1, and *Pantoea* and *Delftia* at S3. The most reactive of the substrates, olivine, strongly facilitated the growth of *Lysinibacillus* at S2 and *Enterobacterales_unclassified* at S3. Surprisingly, the most inert mineral quartz not only led to a sharp increase of *Lysinibacillus* at S2, similar to olivine, but also exhibited a standout positive effect on *Citrobacter* and *Paraclostridium* at S1.

Overall, compared to CK, treatment groups consistently elevated the abundance of Firmicutes, with the highest levels observed under olivine and quartz treatments across all sites. At the genus level, *Bacillus*, a representative of *Firmicutes*, was repeatedly enriched under these two substrates, while other genera such as *Sphingomonas*, *Massilia*, and *Paenibacillus* exhibited site- and treatment-specific responses, showing modest increases under granite and kaolinite at S2 and S3.

### Distribution and diversity of ASVs in treated communities

**Fig 3:**
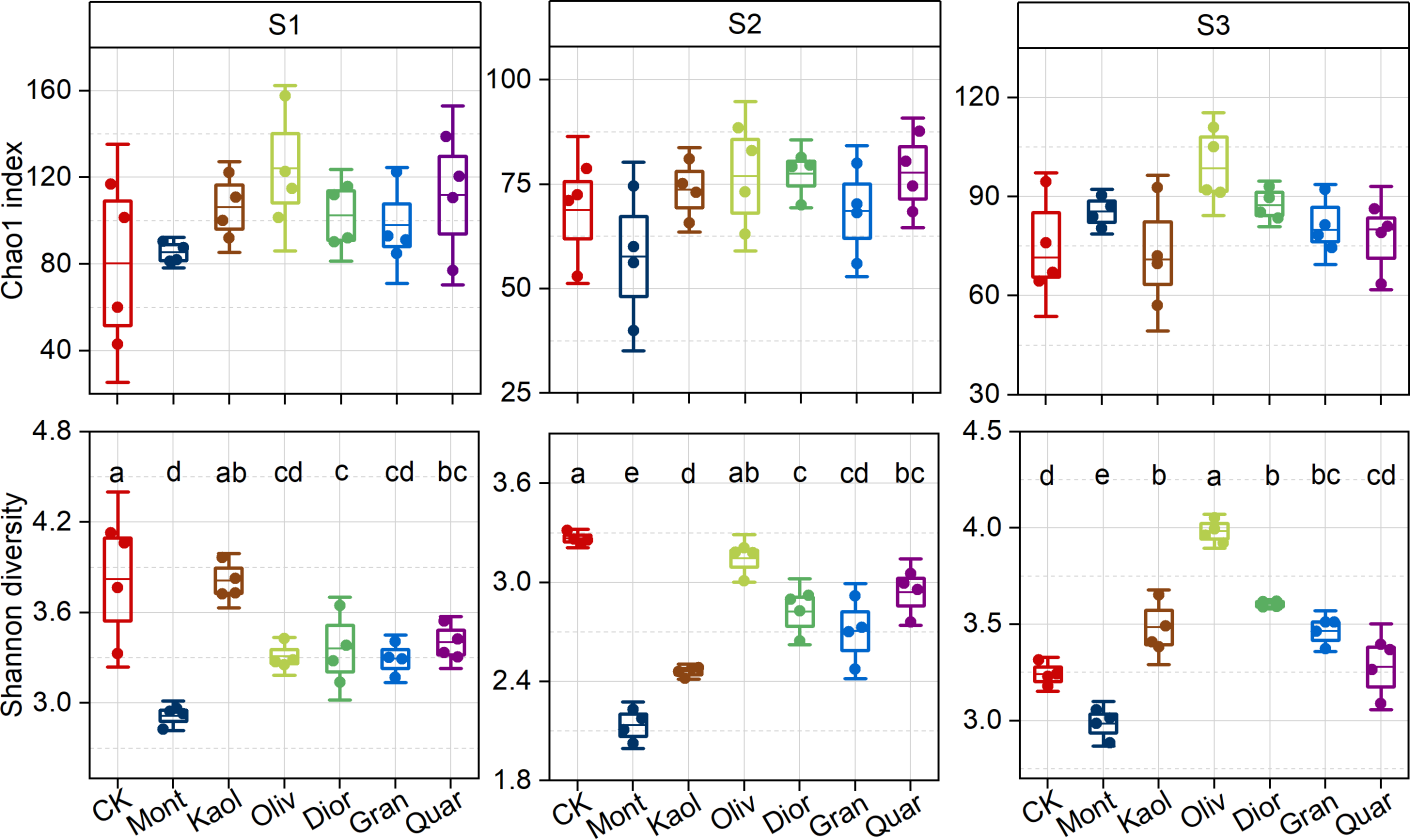
Effects of mineral amendments on the Chao1 richness and Shannon diversity of the bacterial communities after 50 transfers across the three sampling sites. Letters above boxplots indicate significant differences among groups based on Tukey’s HSD test (*P* < 0.05). Data are shown as mean  ±  standard deviation of four independent biological replicates (*n*  =  4).

Chao1 index (Fig. 3) indicated that treatments did not significantly alter species richness. Shannon-Wiener indices (Fig. 3), however, exhibited variation specific to either substrate type or site, as exemplified by the lowest diversity and evenness across all sites under Mont treatment, as well as the highest diversity at S2 and S3 under Oliv treatment and at S1 under Kaol treatment.

**Fig 4:**
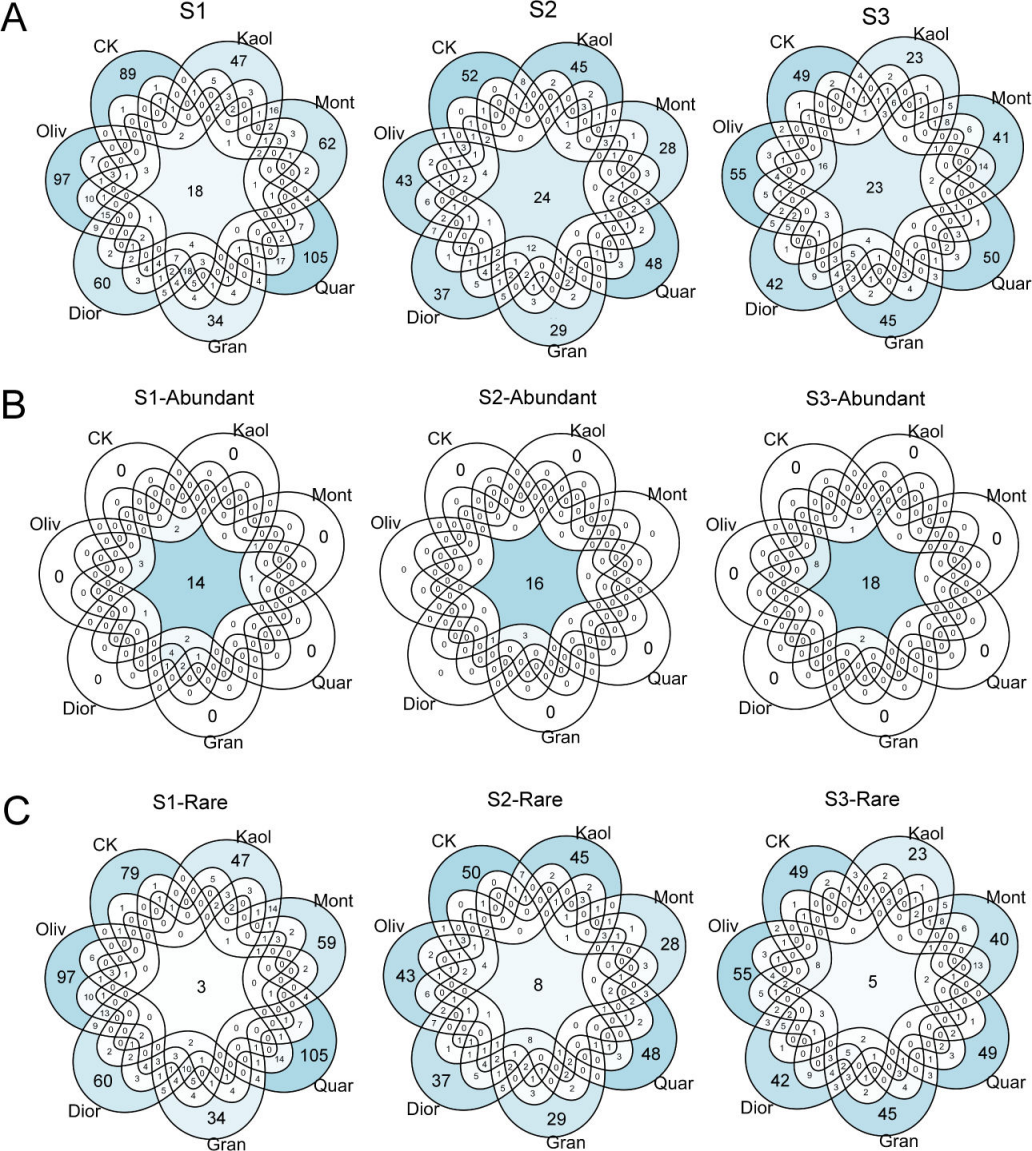
Venn diagram showing sharing distribution of all ASVs (**A**), abundant ASVs (**B**, abundance > 0.1%), and rare ASVs (**C**, abundance < 0.1%) across substrate treatments at the three sites. Note that rare ASVs were highly treatment-specific, while abundant ASVs were widely shared among treatments.

The number of ASVs shared amongst treatments (including control groups) was stable around 21 ± 3 across the three sites (Fig. 4A). However, those unique to substrate varied from 34 to 105 (mean ≈ 70) at S1, 29 to 52 (mean ≈ 40) at S2, and 23 to 55 (mean ≈ 40) at S3 (Fig. 4). In specific, Quar and Oliv consistently showed the highest unique ASV enrichment (105 ASVs, 14.31% of all ASVs), followed by Oliv (97 ASVs, 13.22% of all ASVs), while the substrates associated with the lowest enrichment varied (Gran at S1, Gran and Mont at S2, and Kaol at S3).

Further analysis revealed that the ASVs experienced substrate-specific enrichment were almost exclusively affiliated with rare taxa (abundance <0.1%) (Fig. 4B and C), whereas those shared across treatments were largely abundant taxa (abundance >0.1%). Specifically, the number of commonly enriched ASVs ranged from 15 to 31 per sample among abundant genera, and there was no unique enrichment across treatments. But only 3–8 are rare taxa common to all treatments, unique enrichment across treatments reached a range of 23 to 105 (18.70%–56.43% of all ASVs). For instance, Oliv and Quar consistently enriched the highest number of rare ASVs across the three sites, ranging from 122 to 192 (81.58%–87.14% of all ASVs) among all substrates. And the lowest numbers of unique rare ASVs enriched by substrates were observed under Gran and Kaol treatment at S1 (34 and 47, 22.22% and 29.19%, respectively), Mont and Gran at S2 (28 and 29, 23.97% and 27.18%), and Kaol at S3 (23, 18.70%).

### Treatment-specific enrichment of rare genera

For rare taxa (Fig. S5), montmorillonite promoted the enrichment of several host-associated genera (e.g., *Coprococcus*, *Fusicatenibacter*, *Bifidobacterium*) known for short-chain fatty acid production in host systems, along with environmental taxa (e.g., *Microvirgula*, *Sulfuricurvum*, *Acidibacter*) involved in nitrogen, sulfur, and metal cycling. A few host-associated taxa (e.g., Clostridioides at S2, *Haemophilus* at S3) appeared in limited subgroups. Kaolinite treatment showed a balanced enrichment of host-related taxa (e.g., *Oscillospira*, *Dialister*, *Colidextribacter*) and environmental genera (e.g., *Rhodoplanes*, *Comamonas*, *Desulfovibrio*) involved in carbon, sulfur, and phototrophic metabolism. Olivine-induced enrichment encompassed several taxa commonly detected in human or animal microbiomes (e.g., *Lactobacillus*, *Fusobacterium*, *Escherichia*) as well as environmental bacteria (e.g., *Methylobacterium*, *Brevundimonas*, *Methylibium*) known for pollutant degradation. Diorite favored a mixture of host-associated (*Shigella*, *Escherichia*, *Haemophilus*) and environmental or plant-associated taxa (*Kosakonia*, *Erwinia*, *Actinoplanes*), whereas granite enrichment mainly involved environmental genera (*Sphingomonas*, *Sulfuricurvum*, *Methylocystis*) linked to sulfur, methane, and pollutant cycling. Quartz enrichment displayed a broad functional range, including host-associated (*Roseburia*, *Megamonas*, *Fusobacterium*, *Proteus*) and environmental/agricultural genera (*Bradyrhizobium*, *Rhizobium*) contributing to nitrogen fixation and bioremediation.

## DISCUSSION

### Rocks/minerals as environmental filters for re-structured microbial community

**Fig 5:**
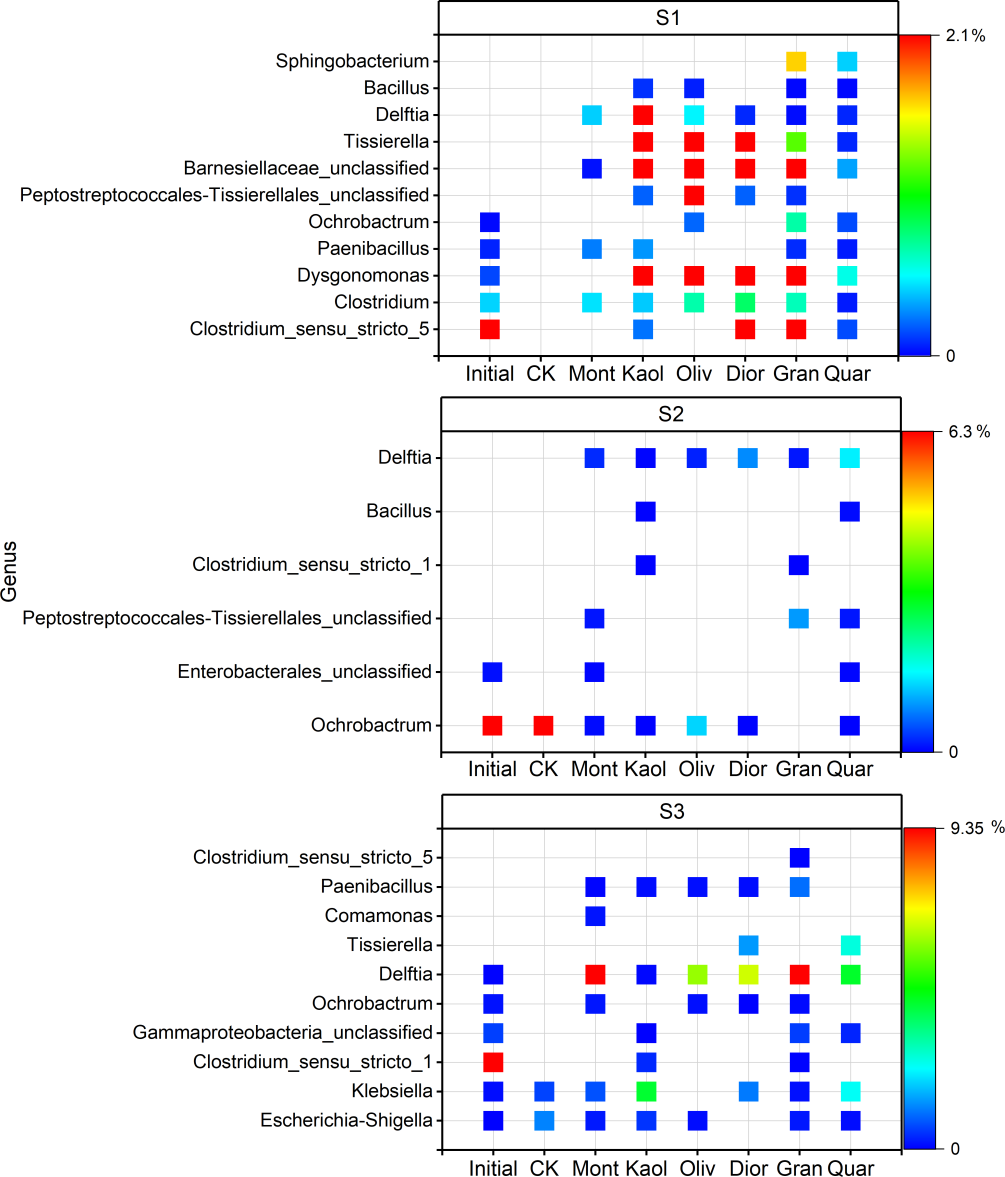
Bubble chart showing the relative abundance of the top 30 most abundant genera in the initial sample (Initial), CK, and six mineral/rock treatments across the three sites. Genera are screened through three methods: (i) lost in CK but enriched in treatments, (ii) retained in CK but diminished in treatments, and (iii) absent in Initial and CK but occurring in treatments. Data are shown as mean of four independent biological replicates (*n*  =  4).

Findings from the serial passage evolution experiments provide strong and consistent evidence that substrate treatments significantly re-structured the soil-derived microbial communities across the three sites, suggesting long-term exposure to substrate may alter the successional trajectory of the consortia. For example, different minerals retain distinct taxa that would have disappeared in the control treatment (e.g., quartz at S3 retained *Clostridium_sensu_stricto*_5), or lead to the formation of new taxa (e.g., kaolinite at S3 enriched *Comamonas*) (Fig. 5). Although a direct correspondence between the growth of individual bacterial taxa and specific substrate properties was not immediately evident, several general functional patterns emerged. Notably, all substrate treatments resulted in an increased relative abundance of *Firmicutes* (Fig. 2A). This shift contributed to an elevated *Firmicutes*/*Bacteroidetes* (F/B) ratio. Although the widely reported link between a high F/B ratio and enhanced energy extraction efficiency in gut microbiomes cannot be directly applied to soil systems, it highlights that the relative abundance of these phyla often reflects differences in substrate use strategies (29). In soils, Firmicutes are often r-selected copiotrophs and fermenters, associated with rapid utilization of labile carbon (30, 31). In contrast, Bacteroidetes frequently harbor specialists in degrading complex polymers like cellulose (32). From this perspective, changes in the F/B ratio may, therefore, indicate shifts in the quality of soil organic carbon—from communities optimized for the rapid turnover of labile C (high F/B) toward communities adapted for decomposing more recalcitrant substrates (low F/B). Furthermore, the selective enrichment of specific taxa by different minerals implies that soil parent material may be underappreciated factors in predicting microbial-mediated ecosystem services. This has practical relevance for land management strategies, where understanding mineral-microbe feedbacks could inform approaches to enhance soil carbon sequestration, improve nutrient cycling efficiency, and maintain agricultural productivity under changing environmental conditions.

Minerals have long been viewed as nutrient reservoirs or colonization scaffolds for microorganisms (12). However, how specific mineralogical traits actively influence microbial community assembly remains insufficiently understood. We hypothesize that properties such as surface structure and charge characteristics, specific surface area (SSA), cation exchange ability, the capacity to mediate microbial extracellular electron transfer (EET), and bulk solubility may serve as environmental filters to deliberately steer microbial colonization and succession (33) similar to micro-growth habitat fragmentation (34). To access this concretely, we compared montmorillonite and kaolinite, two phyllosilicate minerals with distinct surface chemistries, to investigate how these contrasting properties regulate microbial community composition and function.

Montmorillonite, a 2:1 phyllosilicate composed of two tetrahedral SiO_4_ sheets sandwiching one octahedral AlO_6_ sheet. In comparison, kaolinite is a 1:1 phyllosilicate with alternating tetrahedral Si and octahedral Al layers. The structural differences render montmorillonite possessing a higher specific surface area (53.67 vs 10.17 m²/g, Fig. S2) and extensive isomorphic substitution (e.g., Mg²^+^ for Al³^+^ in AlO_6_ and/or Al³^+^ for Si⁴^+^ in SiO_4_). The latter feature not only give montmorillonite more reactive surface sites and composition variety (varying interlayer cations) but greater cation exchange capacity (50–150 vs negligible mmol/kg), structural charges (0.2 to 0.6/half unit-cell vs negligible), and surface acidity (point of zero charge 2 to 3 vs 4.5 to 6.5) (35–37). These characteristics promote strong electrostatic and cation-bridging interactions of montmorillonite with microbial cells, leading to tight cell–mineral binding and the formation of stable inorganic-organic aggregates (Fig. 1). Such environments favor surface-associated, anaerobic taxa like *Clostridium sensu stricto* 13 and *Comamonas* (Fig. 2B), while simultaneously reducing microbial diversity due to niche selectivity. Moreover, montmorillonite’s interlayer structure facilitates adsorption of small organic molecules, including quorum-sensing signals such as C8-HSL, thereby modulating microbial communication and potentially reducing competitive interactions (38). The mineral’s exchangeable cations (e.g. Na^+^, Ca²^+^, Mg²^+^, and even H^+^) may also participate in charge balance to facilitate microbial extracellular electron transfer, further influencing redox-driven microbial processes and favoring biofilm-forming and redox-active taxa. In contrast, the limited reactivity of kaolinite is expected to result in weaker microbial adhesion and hence tends to support communities similar to the control groups (Fig. 1). But compared to control groups, it is characterized by more diverse, planktonic taxa such as *Aeromonas* and *Citrobacter* (Fig. 2), and rare genera like *Dialister* and *Colidextribacter* (Fig. S5). Kaolinite’s poor adsorption of signaling molecules and no redox activity further limit its influence on microbial communication and metabolism. The weak affinity of microbial cells to kaolinite surfaces also explains the absence of strongly surface-bound taxa (e.g., *Clostridium sensu stricto* 13), which are enriched under montmorillonite treatment.

An unforeseen yet interesting case is that of quartz, an inert material to bacteria and expected neutral substrate in the present study. Although quartz occasionally behaved as bystander (e.g., *Acinetobacter* at S1 and S3, Fig. 2B), it nonetheless showed detectable effects on community composition, suggesting that even chemically inert minerals may exert non-negligible influences under prolonged exposure (enriching 32%–50% of unique rare ASVs, Table S2). The most surprising observations came with the nearly quartz-exclusive enrichment of *Pseudomonas*, *Citrobacter,* and *Paraclostridium* at S1 and *Lysinibacillus* (along with olivine treatment) at S2 (Fig. 2B). These findings open several avenues for advancing our understanding of cell-mineral interactions. First, quartz has a moderate solubility with *K*_sp_ = 10^−4^ for SiO_2_ + 2H_2_O = H_4_SiO_4_ (aq). The resultant silicic acid occurs primarily in the form of hydrated SiO_2_ (SiO_2_•2H_2_O) when pH < ~10, forming an amorphous silica gel coating on quartz grains (39). The lack of substrate effect from quartz on bacteria such as *Acinetobacter*, *Betaroteobacteria* unclassified, *Aeromonas* thus may indicate that these organisms, compared to other community members, require either more rigid (i.e., crystalline) or chemically diverse surfaces to gain attachment advantages (Fig. 2B). The same reasoning may apply to quartz-exclusive enrichment of *Pseudomonas* and *Citrobacter* who, among all the community members, thrive on hydrated gel-like surface due to their metabolic versatility, rapid colonization, and ability to form biofilms on inert amorphous surfaces (Fig. 2B) (40, 41). Second, the observation that quartz in most cases was actively influencing community compositions suggests bacterial growth may inherently be sensitive to physical heterogeneity in the environment, particularly in the absence of nutrient stress. While quartz is chemically inert, its role as a physical substrate and microhabitat appears sufficient to selectively favor adaptable colonizers like *Pseudomonas, Citrobacter,* and *Paraclostridium* (Fig. 2B). As bacteria likely have variable surface attachment strategies to gain competitive edges within their communities, the presence of any interfaces, regardless of their chemical properties or reactivities, may provide a physicochemical niche (i.e., environmental filter) favorable to certain opportunists or generalists.

These findings make a strong argument that geological substrates, though abiotic in nature, can play active ecological roles in structuring microbial community. Differences in properties and reactivities of substrate-water interfaces may selectively facilitate/suppress certain taxa and ultimately lead to varied community composition and structure over time. The substrate effects are likely materialized through modifying communication, colonization mode, cell-environment interactions, and ultimately microbial metabolic strategies to impact the long-term community trajectories.

### Mineral-driven selection and adaptive evolution of rare taxa

The observations that taxonomic changes in response to substrate-specific treatments are primarily driven by rare taxa (Fig. 4) suggest these organisms are highly sensitive to the selective pressures imposed by substrates. Rare taxa, typically specialists, fulfill unique ecological roles within the wider community (25–28). It implies that surface-mediated microenvironments promote pronounced niche partitioning, enabling rare taxa (often ecological specialists) to occupy and thrive in unique metabolic roles tailored to specific mineral substrates.

Beyond driving compositional shifts, these enriched rare taxa may putatively expand the functional diversity of the community. While abundant taxa, exhibiting high ASV sharing across treatments, likely form a metabolically versatile core microbiome that sustains baseline functions such as carbon cycling irrespective of mineral context. In contrast, the rare biosphere contributes substrate-specific metabolic capabilities; for instance, montmorillonite was suggested to enrich SCFA producing genera such as *Coprococcus* (Fig. S5) (42), whereas quartz appeared to favor nitrogen-fixing taxa like *Bradyrhizobium* (Fig. S5) (43). This distribution points to a form of functional redundancy, wherein similar ecosystem functions (e.g., micro nutrient cycling) are likely fulfilled by different rare taxa depending on the mineral environment. As environmental conditions fluctuate, whether naturally or through anthropogenic impacts, the diverse metabolic capabilities housed within the rare biosphere provide a buffer against functional collapse. The variety of minerals essentially creates multiple ecological insurance policies, each supporting unique rare taxa that can fulfill similar ecosystem functions through different biochemical pathways. This mineral-mediated functional redundancy may explain why soils with diverse mineralogical compositions often exhibit greater stability in biogeochemical cycling rates and enhanced recovery from disturbances. In the context of ecosystem restoration and soil health management, these findings suggest that preserving mineralogical diversity should be considered alongside traditional biological and chemical soil parameters.

The dichotomous responses of rare and abundant taxa to mineral treatments further illuminate their complementary ecological roles. Mineral substrates consistently elevated the F/B ratio, reflecting the role of abundant taxa (e.g., *Firmicutes*) in supporting core energy metabolism and carbon cycling. In parallel, rare taxa delivered specialized functions, such as sulfur cycling by *Sulfuricurvum* under montmorillonite (Fig. S5), that complement those of core taxa. This functional complementarity, together with the functional redundancy borne by the rare members, enhances the metabolic complexity and ecological resilience, providing alternate metabolic pathways to maintain functioning under changing environmental conditions (44).

In addition to their ecological contributions, mineral-specific enrichment of rare ASVs underscores the role of rare taxa as evolutionary agents within microbial communities. With abundant taxa providing core functions, rare taxa, in light of their high genetic diversity and propensity for horizontal gene transfer (HGT), mutation, and regulatory adaptation, serve as reservoirs of evolutionary innovation (Fig. 5) (45). While not directly explored in this study, our earlier work showed that minerals can act as signaling agents influencing microbial community adaptation, not only altering community structure but also inducing changes in gene expression (33). Moreover, literature data provided evidence of mineral-induced HGT (46) and early-stage microevolutionary divergence or incipient speciation even in the absence of apparent resource limitation (45). Still, recent studies discovered that microbial community composition shift can itself reflect eco-evolutionary dynamics, where ecological selection and genetic diversification operate in tandem (47, 48). Together, our findings and literature evidence support the concept that minerals and rocks, beyond shaping community composition, may drive long-term evolutionary shifts by imposing consistent, substrate-specific selective pressures. As time progresses, such pressure may gradually extend beyond rare taxa, eventually influencing abundant and even dominant members of the community. This trajectory resembles a “bottom-up control” mechanism in ecological trophic cascades, where changes initiated at lower levels propagate upward to restructure the entire system (49, 50).

Collectively, these findings highlight the potential significance of geomaterials as key ecological drivers in soil systems, mediating community structure and function through the selection and enrichment of rare taxa. In turn, by occupying specialized niches, contributing to functional redundancy, and serving as potential agents of adaptation, the rare biosphere extends the ecological reach of mineral–microbe interactions to entire soil system with long-term evolutionary implications. The strong substrate-specific effects on rare taxa suggest that mineral properties may disproportionately influence the evolutionary potential and functional diversity of soil microbiomes. Intensive agricultural practices that homogenize soil mineralogy through parent material mixing or selective erosion may also inadvertently reduce the diversity of mineralosphere-associated rare taxa, potentially diminishing the soil’s adaptive capacity. Conversely, these findings present opportunities for biogeochemical engineering—deliberate introduction or preservation of specific minerals to cultivate desired microbial functions, enhance soil biodiversity, or accelerate ecosystem recovery following degradation

### Conclusions

Through serial passage experiments, we observed that geological substrates, long regarded as passive components of soil matrices, actively restructure microbial communities under nutrient-rich conditions where other environmental factors typically dominate. These geomaterials function as environmental filters that promote substrate-specific assemblages, with particularly pronounced effects on rare taxa abundance and diversity. The consistent enrichment of rare taxa per mineral underscores these organisms’ role in functional redundancy and ecosystem resilience, complementing baseline functions like carbon cycling provided by dominant populations. Moreover, substrate-specific responses of rare taxa suggest rocks and minerals modulate the rare biosphere, a potential seed bank for evolutionary innovation, through genetic diversification under persistent mineral-driven selection.

These results elevate bacterium-mineral/rock interface from a passive zone to a critical microhabitat where abiotic complexity directly governs microbial diversity, function, and long-term evolutionary trajectories. Ultimately, we redefine soil minerals as dynamic architects of microbial community assembly, with broad implications for understanding soil health and ecosystem resilience. These insights not only advance microbial ecology but also offer a mechanistic framework to predict how natural or anthropogenic changes in soil mineralogy—can propagate through microbial communities to influence carbon cycling, agricultural productivity, and ecosystem resilience.

## MATERIALS AND METHODS

### Sampling site and material description

Top soil (0–20 cm) was collected from corn fields at three sites in northeastern China: S1 Shenyang (41°31′17.2″N, 123°22′04.1″E, temperate monsoon climate, mean annual temperature 5°C to 10°C, brown/dark brown soil); S2 Changchun (43°44′37.8″N, 125°22′31.9″E, temperate monsoon, 4.8°C, precipitation 550–700 mm/year, black Mollisols); and S3 Harbin City (45°39′16.4″N, 126°34′07.7″E, mid-temperate continental monsoon, 3.5°C to 5°C with harsh winters of below –30°C, 500–600 mm/year, black Mollisols). A schematic of the sample sites is shown in Fig. S1A. Powdered diorite (Dior), granite (Gran), olivine (Oliv), and quartz (Quar) were acquired from Langfang Mineral Separation Co. (China), and medical grade kaolinite (Kaol) and montmorillonite (Mont) were purchased from Aladdin Chemical Reagent and Hunan Fangsheng Pharmaceutical Co. (both China), respectively. Through SEM (Fig. 1), kaolinite typically exhibits a book-like or stacked lamellar structure, while montmorillonite displays a disordered accumulation of thin, curled, or fringed flakes. Olivine appears as irregular prismatic grains, and diorite presents a blocky structure. Quartz shows larger rock fragments with smooth surfaces, whereas granite exhibits larger rock fragments with more cross-sectional features. The average length of kaolinite, montmorillonite, olivine, and diorite particles is approximately 2–5 μm. Chemical compositions of the samples were determined by X-ray Fluorescence Spectrometer after lithium borate-lithium nitrate fusion (Table S1). Specific surface area was measured by multipoint BET (N2 at 77.35 K) (Fig. S2). Prior to cultivation experiments, minerals were suspended in ultrapure water (Milli-Q, resistivity >18 MΩ·cm) and sonicated for 10 min to remove dust and soluble salts. The suspension was then treated with 0.05 M HCl for 5 min at room temperature, followed by thorough rinsing with Milli-Q water until conductivity and pH stabilized, and autoclaved (121°C, 20 min).

Luria-Bertani (LB) medium (10 g tryptone, 5 g yeast extract, 10 g NaCl L^−1^) was prepared using OXOID tryptone (10 g/L) and yeast extract (5 g/L) in NaCl (10 g/L) solution. HEPES (4-(2-hydroxyethyl)−1-piperazineethanesulfonic acid) was added to buffer pH which was adjusted to 6.8 prior to autoclaving.

### Serial passage evolution experiments setup and cell morphology characterization

Seven groups were established for serially batch cultures in LB medium: a control (CK, sterile distilled deionized water) and six mineral treatments (Mont, Kaol, Oliv, Dior, Gran, and Quar, 10 g/L). Inoculum was prepared by suspending 5 g of soil in 100 mL of sterile shaking (170  rpm for 2 h), settling (10 min), followed by collecting the supernatant. This inoculum underwent three sequential transfers in mineral free LB medium (1% inoculation, ~65 h/cycle, 30°C, 170 rpm) to establish a stabilized “initial community,” which was subsequently inoculated (1% inoculum) into fresh LB media either containing the respective minerals or without minerals (control group) and serially passaged for 50 generations under identical conditions, with quadruplicate per treatment. Final microbial consortia were harvested in each replicate by centrifugation (8,000 rpm at 4°C for 5 min). A schematic diagram of the experimental design for the serial passage culture is shown in Fig. S1B.

The collected cells after 50 passages of cultivation were fixed in 2.5% (vol/vol) glutaraldehyde prepared in phosphate-buffered saline (PBS), dehydrated through a graded ethanol series (20%, 40%, 60%, 80%, 95%, and 100% [vol/vol], 10 min per step), dried, sputter-coated with platinum, and imaged using scanning electron microscopy (SEM; Zeiss Sigma 500). To improve the identification of microbial cells in mineral treatments, cells were color-labeled using Adobe Photoshop software.

### DNA extraction and 16S rRNA gene sequencing

DNA was extracted from the consortium samples using E.Z.N.A. Soil DNA kit (Omega, USA) following manufacturer’s instructions, with concentration and quality verified by NanoDrop and 1% agarose electrophoresis. The 16S rRNA V3–V4 region was amplified using primers 341F (5′-CCTACGGGNGGCWGCAG-3′) and 805R (5′-GACTACHVGGGTATCTAATCC-3′). Each 25 µL PCR contained 12.5 µL PCR premix, 2.5 µL forward primer, 2.5 µL reverse primer, 50 ng template DNA, and nuclease-free water to volume. Thermocycling was 98°C 30 s (pre-denaturation); 35 cycles of 98°C 10 s, 54°C 30 s, 72°C 45 s; followed by 72°C 10 min and a 4°C hold. Amplicons were examined on 2% agarose gels and purified with AMPure XT beads (Beckman Coulter Genomics, USA). Sequencing was performed commercially at LC-Bio (China) on an Illumina NovaSeq 6000 platform (2 × 250 bp).

Raw paired-end reads were demultiplexed by sample barcodes, adapter/primer-trimmed, and quality-filtered (fqtrim v0.94). Read pairs were merged with FLASH, and chimeras were removed with VSEARCH (v2.3.4; UCHIME *de novo*). Length filtering and denoising were performed with the DADA2 algorithm in QIIME2 (v2019.4) to infer amplicon sequence variants (ASVs; 100% identity); singletons were removed. To standardize depth across samples, we rarefied to 44,379 reads per sample; Good’s coverage exceeded 99%. Taxonomy was assigned against the SILVA reference database (release 138), and non-bacterial sequences (chloroplast/mitochondria) were excluded. The resulting ASV feature table (abundances and representative sequences) was used for downstream analyses.

### Statistical analysis

ASVs were classified as “abundant” (mean abundance >0.001 and presence in >50% of samples) or “rare” (<0.001 and/or <10% occurrence) (51–53). Alpha diversity indices (Chao1 richness and Shannon diversity) and Venn diagram analyses were computed in R (v4.3.1) using the vegan and VennDiagram packages. Treatment effect on α diversity and relative abundance was assessed via one-way analysis of variance (ANOVA) in SPSS (v.13.0) and visualized using ggplot2. Bacterial community composition was graphed by Origin 2021.

## Data Availability

The raw sequencing data generated and supplementary data in this study have been uploaded to Figshare (https://doi.org/10.6084/m9.figshare.30069247, https://doi.org/10.6084/m9.figshare.30271837).

## SUPPLEMENTAL MATERIAL

Supplemental material aem.02011-25-s0001.docx Figures S1 to S5; Tables S1 and S2.

ASM does not own the copyrights to Supplemental Material that may be linked to, or accessed through, an article. The authors have granted ASM a non-exclusive, world-wide license to publish the Supplemental Material files. Please contact the corresponding author directly for reuse.
