# Supplementary material for: Mineral substrates as evolutionary drivers of soil microbial diversity through the rare biosphere
Source: Appl Environ Microbiol. 2025 Dec 9;92(1):e02011-25. doi: 10.1128/aem.02011-25 (PMC12838204; doi:10.1128/aem.02011-25)
Supplement: Supplemental material — Figures S1 to S5; Tables S1 and S2. [file aem.02011-25-s0001.docx]

**Mineral Substrates as Evolutionary Drivers of Soil Microbial Diversity Through the Rare Biosphere**

Beibei Wang, Jianchao Zhang*, Xiangyu Zhu, Yuebo Wang, H. Henry Teng*

School of Earth System Science, Institute of Surface-Earth System Science, Tianjin University, Tianjin, 300072, China

*Corresponding author:

Jianchao Zhang: jianchaozhang@tju.edu.cn

H. Henry Teng: hteng@gwu.edu


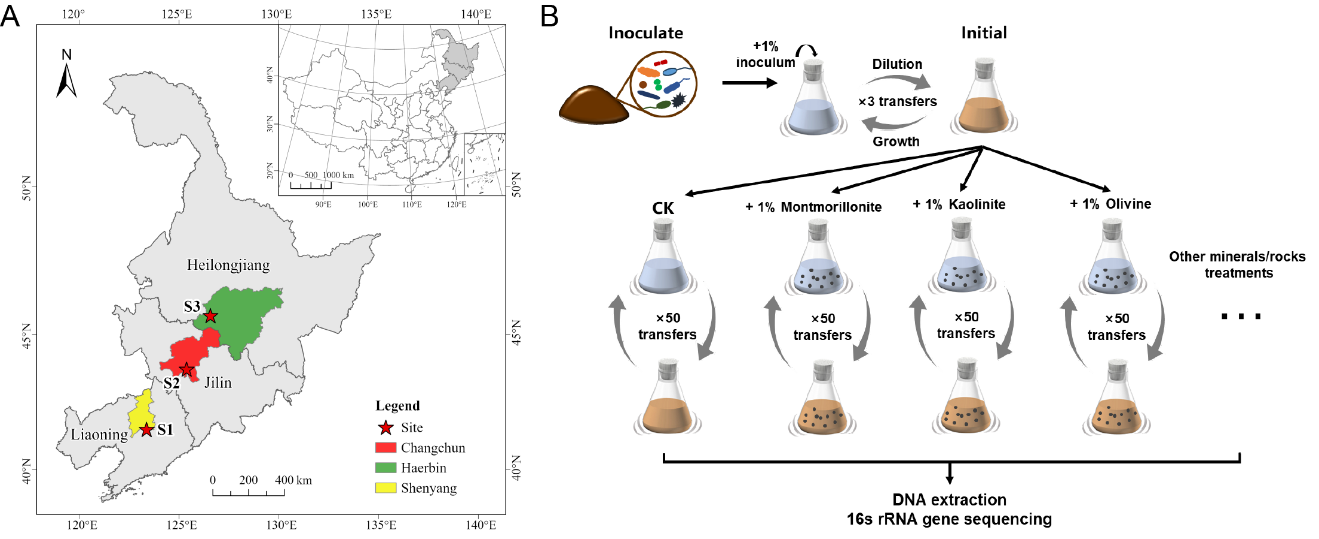


Fig. S1. (A): Schematic diagram of the sampling site. (B): Schematics of the serial passage evolution experiment workflow. The ‘initial’ community was established from the original soil solution inoculant after three sequential transfers, which was used subsequently to inoculate the substrate-containing media for treatment experiments.

.


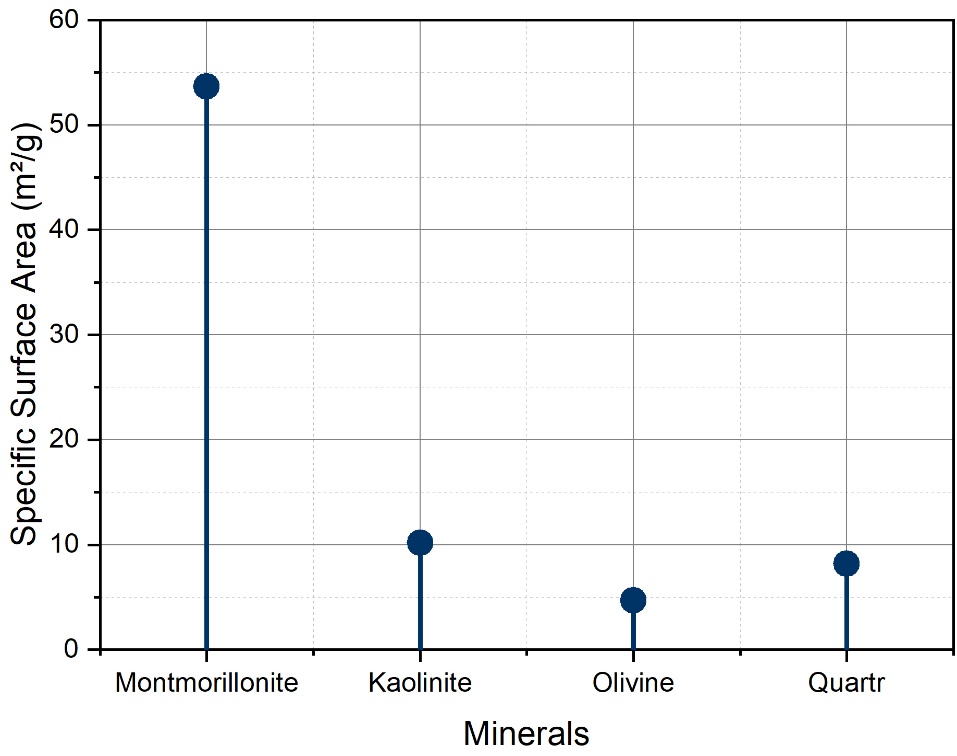


Fig. S2. Specific surface area of different minerals (Olivine, Quartz, Kaolinite, Montmorillonite) measured by the multipoint BET method (N_2_ adsorption at 77.35 K). Data are presented in m²/g, reflecting the surface physical properties of each mineral sample.


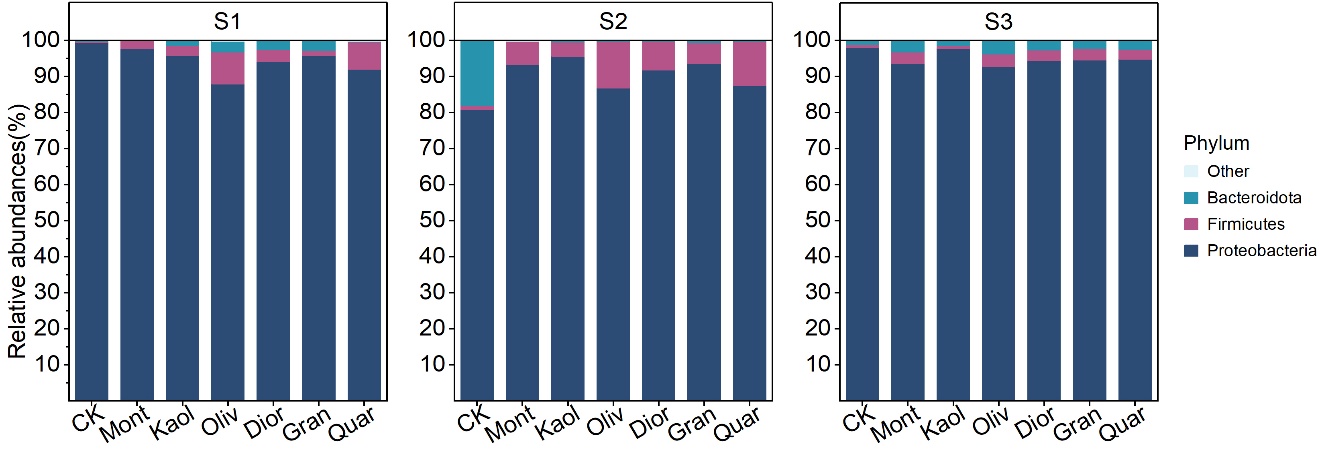


Fig. S3. Stacked bar charts showing relative abundance of bacterial phyla across sites and treatments. Note the consistent dominance of *Proteobacteria* in all cases.


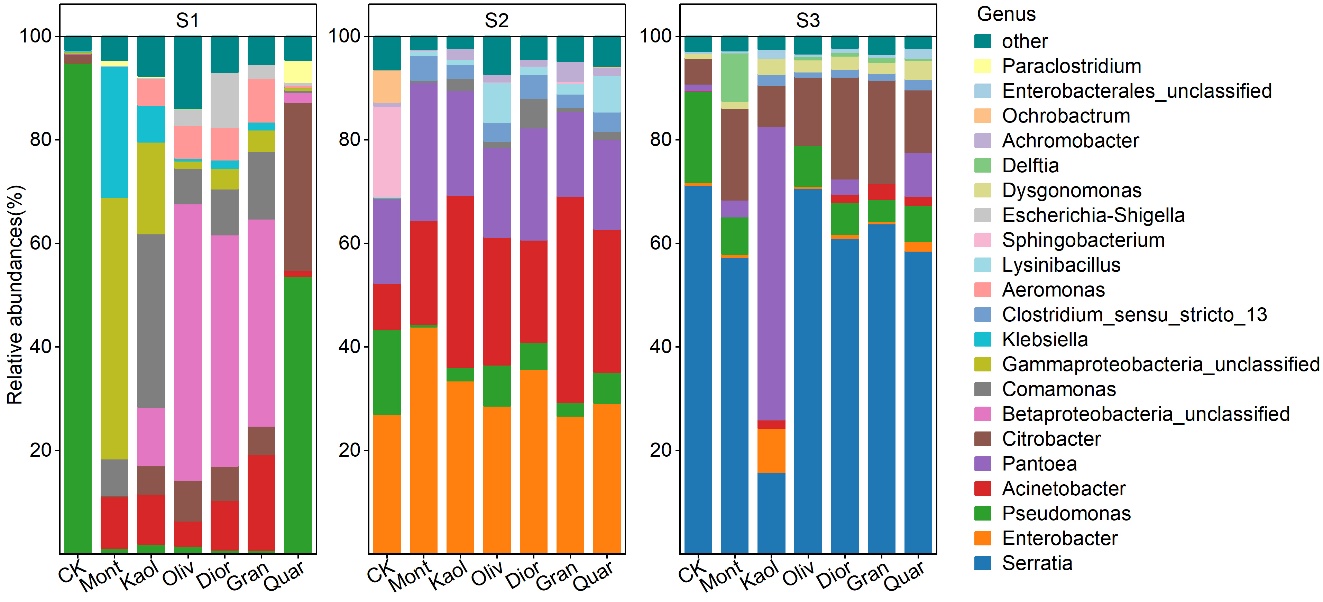


Fig. S4. Stacked bar charts showing relative abundance of bacterial genera across sites and treatments.


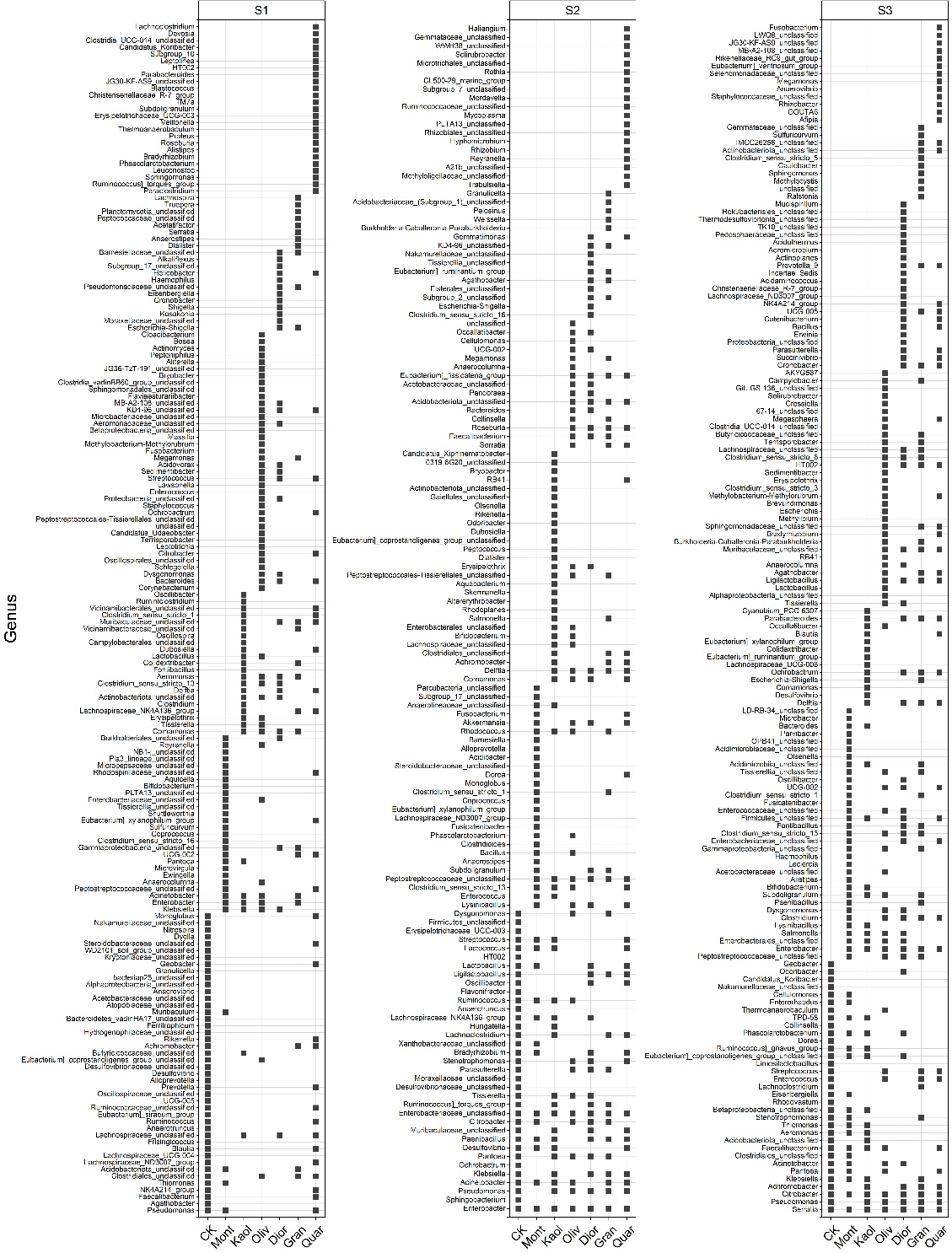


Fig. S5. Distribution of rare bacterial genera across different mineral/rock treatments showing the treatment-specific enrichment patterns of rare taxa at the sampling sites. Each black square indicates the presence of a specific rare genus under a given treatment condition. Only genera with relative abundances below 0.1% across all samples are included.

**Table S1. Chemical Composition of Minerals and Rocks**

|  | **TFe_2_O_3_** | **MgO** | **SiO_2_** | **Al_2_O_3_** | **CaO** | **K_2_O** | **Na_2_O** | **Formula^*^** |
| --- | --- | --- | --- | --- | --- | --- | --- | --- |
| **Montmorillonite (%)** | 8.84 | 46.1 | 43.03 | 0.24 | 0.4 | 0 | 0.1 | (Na,Ca)₀.₃(Al,Mg)₂,Si₄O₁₀(OH)₂·nH₂O |
| **Kaolinite (%)** | 0.59 | 0.08 | 46.01 | 38.28 | 0.02 | 0.12 | 0.07 | Al₂Si₂O₅(OH)₄ |
| **Olivine (%)** | 1.5 | 0.07 | 76.94 | 12.23 | 0.28 | 4.49 | 3.77 | (Mg,Fe)₂SiO₄ |
| **Diorite (%)** | 1.1 | 0.01 | 97.79 | 0.3 | 0.1 | 0.13 | 0 | Na₀.₅Ca₀.₅Al₁.₅Si₂.₅O₈, Ca₂(Mg,Fe,Al)₅, (Si,Al)₈O₂₂(OH)₂ |
| **Granite (%)** | 3.46 | 3.48 | 67 | 13.13 | 1.96 | 0.19 | 0.16 | SiO₂, KAlSi₃O₈, NaAlSi₃O₈ |
| **Quartr (%)** | 7.34 | 2.97 | 56.97 | 17.96 | 5.87 | 1.8 | 3.63 | SiO₂ |

*: Molecular formulas are estimated based on chemical component

Table S2. Quantitative summary of overall, abundant, and rare ASVs across treatments. The table presents the total number of ASVs, the number and proportion of abundant and rare ASVs (%), as well as the number and proportion of unique rare ASVs (%) contributing to overall community diversity.

|  | All ASV | Abundant ASV | Rare ASV | Rare ASV Proportion (%) | Unique Rare ASV | Unique Rare ASV Proportion (%) |
| --- | --- | --- | --- | --- | --- | --- |
| **CK** | 140, 119, 148 | 22, 16, 29 | 118, 103, 119 | 84.29, 86.55, 80.40 | 79, 50, 49 | 56.43, 42.02, 33.11 |
| **Mont** | 135, 103, 136 | 22, 20, 23 | 113, 83, 113 | 83.70, 80.58, 83.09 | 59, 28, 40 | 43.70, 27.18, 29.41 |
| **Kaol** | 161, 135, 123 | 31, 19, 31 | 130, 116, 92 | 80.75, 85.93, 74.80 | 47, 45, 23 | 29.19, 33.33, 18.70 |
| **Oliv** | 223, 142, 171 | 31, 20, 31 | 192, 122, 140 | 86.10, 85.92, 81.87 | 97, 43, 55 | 43.50, 30.28, 32.16 |
| **Dior** | 175, 141, 140 | 30, 20, 31 | 145, 121, 109 | 82.86, 85.82, 77.86 | 60, 37, 42 | 34.29, 26.24, 30.00 |
| **Gran** | 153, 121, 154 | 31, 20, 29 | 122, 101,125 | 79.74, 83.47, 81.17 | 34, 29, 45 | 22.22, 23.97, 29.22 |
| **Quar** | 210, 150, 152 | 27, 20, 28 | 183, 130, 124 | 87.14, 86.67, 81.58 | 105, 48, 49 | 50.00, 32.00, 32.24 |
